# Evaluation of major historical ICR cell designs using electric field simulations

**DOI:** 10.1002/mas.21671

**Published:** 2020-11-25

**Authors:** Evgeny Nikolaev, Anton Lioznov

**Affiliations:** ^1^ Center for Computational and Data‐Intensive Science and Engineering Skolkovo Institute of Science and Technology Moscow Russia

**Keywords:** FT‐ICR, ICR cell, ion cyclotron resonance, ion traps, MS, resolving power

## Abstract

In Fourier‐transform ion cyclotron resonance mass spectrometry, ions are detected by measuring image current induced in the detecting electrodes by trapped ions rotating in a magnetic field at their cyclotron frequencies. The ion trap used for this purpose is called the Penning trap. It can have various configurations of electrodes that are used to create a trapping electric field, to excite cyclotron motion, and to detect the induced signal. The evolution of this type of mass spectrometry is mainly driven by progress in the technology of superconducting magnets and in the constantly improved design of the ion cyclotron resonance (ICR) measuring cell. In this review, we focus on ICR cell designs. We consider that the driving forces of this evolution are the desire to increase resolution, mass accuracy and dynamic range, as well as to adapt new methods for creating and trapping ions.

## 1 INTRODUCTION

In the ion cyclotron resonance method, mass spectra are obtained by measuring the frequency of ion rotation in a magnetic field. Ions are trapped in a homogeneous magnetic field in an ion trap which creates a trapping electric field along the magnetic field lines. Such a trap is called the Penning trap and was invented to hold plasma in the 1930s (Penning, 1936). To excite rotational (cyclotron) motion in such a trap, two electrodes are used, which create an RF electric field perpendicular to the magnetic field with a frequency, equal to that of the ion rotation in the magnetic field, called the “cyclotron frequency.” The signal from the rotating ions can be measured, in the simplest case, using another pair of electrodes, on which rotating ions induce an electric potential. Frequency analysis of the signal is performed with Fourier transform. Such an ion trap is a measuring device, called traditionally in the Fourier‐transform ion cyclotron resonance (FT‐ICR) mass spectrometry community as the “ICR cell” (in this article we will use the terms “ion cell” and “ion trap” as synonyms).

Apart from the evolution of electronics and computing power over almost 50 years of the method's existence (since 1974 (Comisarow & Marshall, 1974)), we can say that the performance improvement of the FT‐ICR technique is mainly associated with the increase in the magnetic field—from 2‐T electromagnets in the first FT‐ICR spectrometers to 21 T in the cutting‐edge modern devices—and with the changes in the design of ICR cells. The transition from production of ions inside the cell (mainly by electron impact and laser ablation ionization) to their introduction from external ion sources significantly changed the FT‐ICR instrument design in general, but did not cause much changes in the cell design (no changes at all in case of collision assisted or gated trapping and only a few changes in the design of the front trapping plate in case of “side kick” trapping). By changing the design of the cells, researchers pursued several goals: increase in resolution of the device, accuracy of mass measurement, and dynamic range of different *m*/*q* (mass to charge) ions.

The first stages of FT‐ICR evolution were caused by the transition from electromagnets with small (about 2 in. maximum) gaps between the poles to a superconducting solenoid with a cylindrical “warm” tube along the axis of the solenoid (room temperature bore) with a diameter of about 5 in. Such a change in the configuration of the magnets caused a conversion from the cubic geometry of the cells to a cylindrical one and an increase in the length of the cell. This transition took a surprisingly long time. After the introduction of solenoidal superconducting magnets (Alleman et al., 1980), more than half of the commercial cells were still either cubic (Extrel) or rectangular (IonSpec). The cylindrical cell was eventually used in Bruker instruments but did not gain popularity in academic FT‐ICR labs or with the other vendors until the mid to late 90s.

The next significant change in the design of the cells was caused by the awareness of the need to make the amplitude of the radiofrequency (RF) electric field which excites cyclotron motion, more uniform along the magnetic field direction in the whole volume of the trap. Its inhomogeneity in the first traps led to the excitation of ions with different axial oscillation amplitudes to different cyclotron radii. In addition, axial oscillations excited by the axial component of such field along with cyclotron motion lead to the ejection of ions from the cell.

The purpose of recent changes in the design of FT‐ICR cells was to further increase the resolution of the device and the dynamic range to fulfill the needs for the analysis of complex mixtures (oil, humic substances), containing hundreds of thousands of components with concentration differences of several orders of magnitude (Mullins et al., 2007). The configuration of the trapping fields in the cells at that time was not ideal. Supercomputer simulations of motion of ionic ensembles inside FT‐ICR cells with such nonideal field showed that the ensembles themselves change their shape during the measurement cycle from elliptical clouds to comet‐like shapes and then to a rotating cylinder, which does not induce any signal on the detection electrodes. The formation of comets in such traps is caused by the dependence of the cyclotron motion frequency (effective frequency) on the amplitude of axial oscillations (for ions excited to the same cyclotron radius). It became clear that such effects can only be eliminated by creating a trap field inside the cell similar to that of a hyperbolic cell, in which all three modes of ion motion in the trap—cyclotron, magnetron, and axial—are separated. The design of the most modern cells with this type of electric field prevents comet formation (Boldin & Nikolaev, 2011). Thus, such traps allow to work at high (up to 10 V) trapping potentials and to hold up to 10^7^ ions in the trap.

In the last decade, a lot of attention was paid to the harmonics in FT‐ICR spectra, which are caused by geometric factors—odd harmonics are the result of flattening of nonideally cosine time‐domain signals from ions with large cyclotron radii and even harmonics are caused by magnetron motion in cases when the center of ion motion does not coincide with the center of the trapping electric field. The presence of harmonics in spectra complicates their interpretation and should be reduced by more accurate cell assembly and electrode production. In contrast, harmonics can be used to increase the resolving power and special cell designs were offered to detect signals at higher harmonics of the cyclotron frequency.

Several reviews devoted to the evolution of ICR cell designs were published earlier. Among them, Guan and Marshall (1995) and Marshall et al. (1998) reviewed the ion traps existing at those times. Also, the recent book *Fundamentals and Applications of Fourier Transform Mass Spectrometry* (Schmitt‐Kopplin & Kanawati, 2019) includes a brief history of FT‐ICR cells. Although the first two articles were comprehensive and well‐written, they are at least two decades out of date in FT‐ICR evolution, while the third one does not focus on the trap design.

We are grateful for the opportunity to devote this article to Alan Marshall—coinventor of FT‐ICR mass spectrometry (Comisarow & Marshall, 1974). Marshall's group made a significant influence on the FT‐ICR mass spectrometry development since its invention. They have developed the cubic trap (Comisarow & Marshall, 1974), the screened trap (Wang & Marshall, 1989), matrix‐shimmed trap (Jackson et al., 1999), and many others. They have developed different excitation modes, for example, SWIFT excitation (T. C. L. Wang et al., 1986). Many, if not all, questions, regarding the improvement of the method, were either asked by the Marshall's group, proposed, or resolved by the group. Also, Marshall's group has always responded to innovations offered by other groups by implementing them in their instruments, as for example, was the case with the compensating traps and traps with dynamic harmonization (Moore et al., 2018).

Alan Marshall wrote several comprehensive reviews of the area, which were also cited in this article. The purpose of our review is not to describe all ion traps created under the supervision of Alan Marshall or all FT‐ICR traps in general, but rather to focus on the traps that (in our opinion) had the greatest influence on the development of the method and understanding of the physics of the measurement procedure. For the description of these cells, we have proposed and implemented a unified approach to explain the properties of various traps that arose during the evolution of the ICR method in response to its needs.

## 2 THE FUNDAMENTALS OF FT‐ICR

The basis of the ICR method has been described in many papers and books. Besides in this specific issue, many articles also explain the basics of the FT‐ICR technique but for consistency, we will include some introductory remarks on this subject.

In the case of a spatially and temporally homogeneous magnetic field *B*, an ion of mass *m* and charge *q* with a nonzero kinetic energy and momentum components perpendicular to the magnetic field will rotate with a constant frequency called the “(pure) cyclotron frequency.”

(1) $$\omega_{c}=\frac{q}{m}B.$$

Note, that in the absence of an electric field, the frequency depends on neither the radius of the orbit nor ion energy.

Ions experiencing the action of only a homogeneous magnetic field could not be confined along the field (also on the plane perpendicular to the field). To trap ions, an electric potential barrier is applied in the direction along the magnetic field. This is usually done either by flat “end‐cap” electrodes used in the cells of the first generations or with cylindrical trapping electrodes in the so‐called “open cells.” In the next few paragraphs, we will briefly describe the influence of these trapping fields on ion motion.

An FT‐ICR mass spectrometer usually deals not with a single ion, but rather with a large number of ions and ionic ensembles of different *m*/*q*. In this case, it is necessary to create an electric field that will keep the ions with the same *m*/*q* in compact ion clouds to produce a signal. To prevent ion cloud degradation (dephasing, known as “comet formation”), the motion in the *x,y* plane must be independent of the motion along the *z* axis (and the frequency of motion in the *x,y* plane must be also independent of cyclotron radius if ions are excited to different radii). If such a dependency is present, the cloud will form a comet, as will be shown below. Here and below, we will consider the *z* axis to be codirected along the magnetic field *B*, so the *x,y* plane is considered to be perpendicular to *B*. It can also be shown that formally the motion in the *x,y* plane is independent of *z* only if the Laplacian of the electric potential Δ*ϕ *=* d* ^2^ *ϕ*/*dx* ^2^ + *d* ^2^ *ϕ*/*dy* ^2^ + *d* ^2^ *ϕ*/*dz* ^2^ does not depend on *z*, so *ϕ*(*z*) ~ *z* ^2^.

The full expression of the simplest potential that has a *z* ^2^ dependency on *z* and satisfies the Laplace equation is (Guan & Marshall, 1995) as follows:

(2) $$\varphi \left(x,y,z\right)=\alpha \left(z^{2}-\frac{x^{2}+y^{2}}{2}\right)+\chi ,$$

where *α* and *χ* are coefficients determined by the voltages on the electrodes and the trap geometry parameters. In the text, we will call such a potential “harmonic” or “ideal.” Note, that *z* ^2^ − (*x* ^2^ + *y* ^2^)/2 equals to *r* ^2^ *Y*_20_ with some coefficient, where *r* is the radius vector to the point and *Y*_20_ is a spherical harmonic (see below for details).

The problem of how to build an ion trap, whose electrodes would produce a harmonic potential is complicated. A big part of the FT‐ICR method evolution was about creating such kind of traps. One of the solutions is to add to a regular FT‐ICR cell some extra—so‐called “compensating”—electrodes, which would make the potential closer to the ideal one.

The appearance of a static trapping electric field component *E* in the plane perpendicular to the magnetic field leads to a mode of slow motion, which is called the “magnetron motion.” The magnetron motion describes the motion of the center of the cyclotron motion trajectory moving in the *x,y* plane in the direction perpendicular to both the magnetic and radial electric fields (along electric field equipotentials).

The radial component of the force *F* acting on any particular ion is

(3) $$F_{\rho }=m\omega^{2}\rho =F_{\mathrm{magnetic}}-F_{\mathrm{electric}}\left(\rho \right)=\mathrm{qB}\omega \rho -\mathrm{qE}(\rho ),$$

where *ρ *=* *(*x* ^2^ + *y* ^2^)^1/2^. By solving the equation for *ω* we get (Marshall et al. 1998; Nikolaev et al., 2019) in common as follows:

(4) $$\omega_{\pm }=\frac{\mathrm{qB}}{2m}\pm \sqrt{{\left(\frac{\mathrm{qB}}{2m}\right)}^{2}-\frac{\mathrm{qE}(\rho )}{m\rho }}.$$

And in case of an ideal field (2):

(5) $$\omega_{\pm }=\frac{\omega_{c}}{2}\pm \sqrt{{\left(\frac{\omega_{c}}{2}\right)}^{2}-\frac{q\alpha }{m}}=\frac{\omega_{c}}{2}\pm \sqrt{{\left(\frac{\omega_{c}}{2}\right)}^{2}-\frac{\omega_{z}^{2}}{m}},$$

where *ω*_+_ is called the “reduced” cyclotron frequency, *ω*_−_ is the “magnetron” frequency, and *ω*_z_ = (*2qα/m*)^1/2^ is the axial oscillation frequency.

These frequencies are connected by the following relations: 2*ω*_+_ × *ω*_−_ = *ω* ^2^_z_, *ω*_c_^2^ = *ω*_+_^2^ + *ω*_−_^2^ + *ω*_z_^2^, as can be obtained from Equation (5). Usually, *ω*_c_ ≳ *ω*_+_ ≫ *ω*_z_ ≫ *ω*_−_. Also, with the help of the Taylor series decomposition, it can be shown from the same equation, that the magnetron frequency depends on magnetic field *B* as *ω*_−_ ~ 1/B. As shown in Jertz et al. (2015), the magnetron motion provides additional harmonics in the spectrum.

### 2.1 Effects of the nonideality of the electric field distribution: Comet formation

As follows from Equation (5), both magnetron and reduced cyclotron frequencies are independent of *z* and *ρ*. But in cases when the electric field distribution does not exactly satisfy Equation (2), this kind of dependence takes place.

To analyze the dependence of frequencies on the ion locations in a trap (especially from the *z* coordinate which has the biggest impact), it is useful to look at the electrostatic potential in spherical harmonics decomposition (Barlow & Tinkle, 2002, 2006; Van Dyck et al., 1986).

(6) $$\phi (r,\theta ,\varphi )=\sum_{l=0}^{\infty }\sum_{m=-l}^{l}A_{\mathrm{lm}}Y_{\mathrm{lm}}\left(\theta ,\varphi \right)\times r^{l}.$$

The spherical harmonics are smooth functions representing the orthonormal basis of functions on the sphere (in other words—the functions from two arguments—polar and azimuthal angles). This means, in particular, that any function on a sphere can be presented as a sum of spherical harmonics. (By analogy, any function from one variable can be presented as a Fourier series (sum of cos and sin functions).

As it was shown in Nikolaev et al. (2016), the field can be approximated quite accurately by the first terms of this sum *A*_20_ *r* ^2^ *Y*_20_, *A*_30_ *r* ^3^ *Y*_30_, *A*_40_ *r* ^4^ *Y*_40_ in the whole working volume. We will use a slightly different approximation in our paper. Practically, all traps have reflection symmetry (across the *x,y* plane), so all odd field harmonics, which do not have such a symmetry, are zeroed out. Since generally the *Y*_40_ harmonic has the biggest side impact on the ion motion, some traps are built so as to nullify the *A*_40_ coefficient. That is why it may be useful to take into account also the *Y*_60_ harmonic. So, for an adequate approximation, the final set should contain the following terms: *A*_00_ *Y*_00_, *A*_20_ *r* ^2^ *Y*_20_, *A*_40_ *r* ^4^ *Y*_40_, *A*_60_ *r* ^6^ *Y*_60_. *A*_00_ *Y*_00_ is a constant, so in the equation below, we will use variable *C* instead of it.

(7) $$\begin{array}{l} \phi \left(r,z\right)≃A_{20}\left(z^{2}-\frac{\rho^{2}}{2}\right)+A_{40}\left(8z^{4}-24z^{2}\rho^{2}+3\rho^{4}\right)\\ \;+A_{60}\left(16z^{6}-120z^{4}\rho^{2}+90z^{2}\rho^{4}-5\rho^{6}\right)+C, \end{array}$$

(8) $$\begin{array}{l} E\left(\rho \right)=-\frac{\partial \phi }{\partial \rho }=A_{20}\rho +A_{40}(48\rho z^{2}-12\rho^{3})\\ \;+A_{60}(240z^{4}\rho -360z^{2}\rho^{3}+30\rho^{5}). \end{array}$$

Thus, from Equation (4) we have

(9) $$\begin{array}{l} \omega_{+}\left(\rho ,z\right)=\frac{\mathrm{qB}}{2m}+[{\left(\frac{\mathrm{qB}}{2m}\right)}^{2}-\frac{q}{m}\{A_{20}+A_{40}(48z^{2}-12\rho^{2})\\ \;{+A_{60}(240z^{4}-360z^{2}\rho^{2}+30\rho^{4})\}]}^{1/2}. \end{array}$$

**Figure 1:**
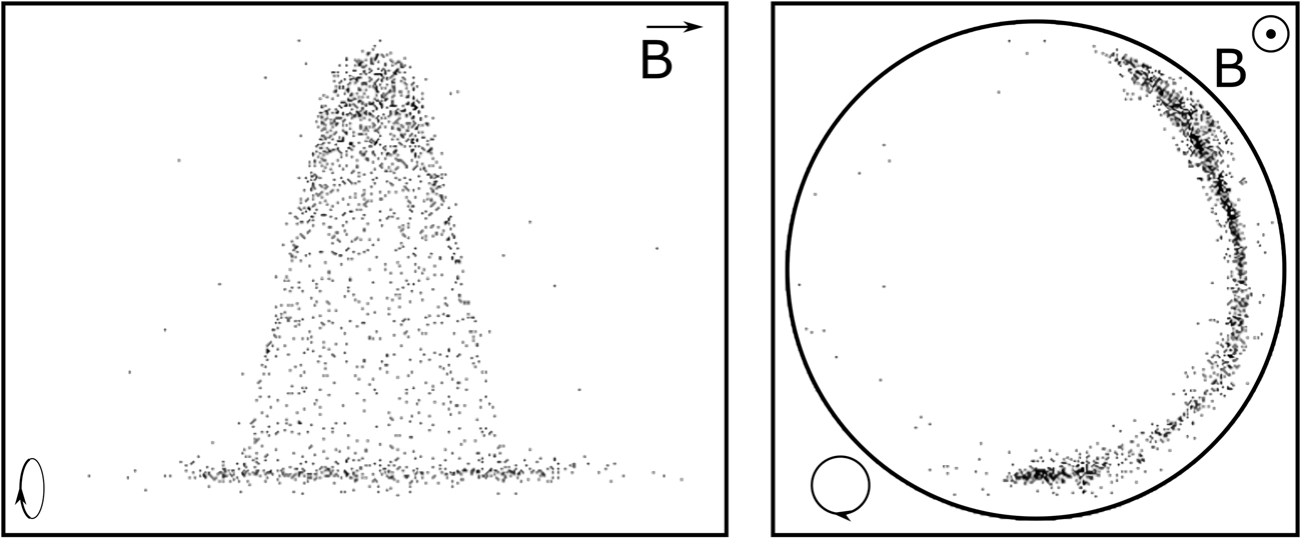
Comet formation inside the Fourier‐transform ion cyclotron resonance cell. The picture of the comet of single *m*/*q* ions in two projections: in *z,y* plane (left) and *x,y* plane (right). The supercomputer simulation was carried out with the particle‐in‐cell code (Nikolaev et al., 2007)

Ion motion in the case when *ω*_+_ depends on (*ρ*, *z*) leads to comet formation, as it was shown in Nikolaev et al. (2007). An initially elliptical ion cloud becomes distributed and is converted into a rotating charged cylinder sometime after excitation; thus the induced potential on the detection electrodes becomes constant and the signal collapses (Figure 1).

### 2.2 Excitation

To detect the ions, they must be excited to a significantly large radius (usually approximately half of the cell radius). Besides this, excitation is used to throw the unwanted ions out of the trap to isolate the ions of interest or to increase the kinetic energy for ion dissociation in case of in cell collision‐induced dissociation and for investigation of ion–molecule reaction kinetics and thermodynamics (Marshall et al., 1998).

The excitation of ions in the FT‐ICR cells was described and analyzed in numerous papers, many of which came from the Marshall's group (Guan & Marshall, 1995; Marshall et al., 1998). In the majority of cell types, the dipolar excitation mode is used. Frequency‐sweep (“chirp”) or, in many cases, the so‐called Stored Waveform Inverse Fourier Transform excitation or *SWIFT* (Cody et al., 1987; Guan & Marshall, 1996; T. C. L. Wang et al., 1986) methods are used to excite ions in a broad mass range. These methods allow ions of different *m*/*q* to be excited in parallel. In hyperbolic cells, this excitation mode can be applied as well, but, for this purpose, the central toroidal electrode should be split into two. In the case of multiple electrode cells (see below), quadrupolar and multipolar excitation modes are also possible and described in Guan and Marshall (1995), Jackson et al. (1997), and Schweikhard et al. (1992) but, these methods have not become widely used in analytical FT‐ICR MS yet.

Nowadays, for cells with multielectrode detection, it is possible to install fast switches permitting to use the same electrodes both for excitation and detection (see below).

### 2.3 Detection

The theory of signal detection from ions with excited cyclotron motion was also discussed in many original publications and reviews. To explain harmonics suppression and multielectrode detection, general signal detection mechanisms are discussed here too.

The theory of signal detection is mainly based on the *reciprocity principle*.

This principle (Dunbar, 1984; Guan & Marshall, 1995; Landau et al., 2013) claims the following: To calculate the charge *Q*, induced on grounded electrodes by an ion with a charge *q* at some position, first, the potential *ϕ* in the position of this ion, created by these electrodes under some constant potential applied to them (say 1 V) while keeping other electrodes grounded must be calculated. After this, *Q* can be obtained by a simple Equation (10).

(10) Q=−qϕ.

A signal is obtained from time‐varying charges on the detection electrodes, induced by ions. First, the induced current is measured

(11) $$J=\frac{\mathrm{dQ}}{\mathrm{dt}}=-q\frac{d\phi }{\mathrm{dt}},$$

and then, the voltage difference (proportional to Δ*Q*) on the detection electrodes caused by the induced or image current across the detection resistor can be recorded. Because of the *superposition principle*, the current and, thus, the signal from multiple ions can be calculated by the sum of the contributions of each ion. Thus, all ions can be detected simultaneously.

The theory of ion detection in specific traps was developed along with these traps. A simple case, for which the analytical solution could be easily obtained, is an infinitely long cylindrical cell. For this trap (Nikolaev & Gorshkov 1985; Nikolaev et al., 2019), the solution for *ϕ*(*ρ*, *φ*) and for charge difference on the detection electrodes is

(12) $$\phi =\frac{\alpha }{\pi }+\sum_{n}\frac{\rho^{n}}{R^{n}}\frac{\sin n\alpha \times \cos n\beta }{n\pi }\cos n\varphi ,$$

(13) $$\Delta Q\sim -q\left(\sum_{n=0}^{\infty }{\left(\frac{\rho }{R}\right)}^{2n+1}\frac{2\sin\left(\left(2n+1\right)\alpha \right)}{\left(2n+1\right)\pi }\cos\left(\left(2n+1\right)\varphi \right)\right).$$

The time profile of the signal can be obtained from Equation (13) with parametrization *ρ *=* ρ*(*t*) and *φ *=* φ*(*t*). This simple approximation can help understand the presence of harmonics in the signal.

#### 2.3.1 Harmonics in case of cyclotron motion

In the case when only cyclotron motion is excited *φ* = *ω*_+_ *t*, *ρ* = const the signal can be calculated from Equation (13) as follows:

(14) $$V\left(t\right)\sim \left(\sum_{n=0}^{\infty }{\left(\frac{\rho }{R}\right)}^{2n+1}\frac{2\sin\left(\left(2n+1\right)\alpha \right)}{\left(2n+1\right)\pi }\cos\left(\left(2n+1\right)\omega_{+}t\right)\right).$$

From this equation, it is clear that the signal contains not only the main frequency *ω*_+_ but also odd harmonics: 3*ω*_+_, 5*ω*_+_, … with amplitude ratio V_2*n *− 1_/V_2*n* + 1_ = (ρ/*R*)^−2^ + O((ρ/*R*)^−^^2^).

The third harmonic provides the biggest side effect on the spectrum. It becomes a problem in complicated multiple *m*/*q* spectra, because in such spectra it is unclear whether a peak arises because of the presence of ions with *m*/*q* corresponding to this frequency or is it just a harmonic induced by another ion. Several papers discuss the ways to reduce the amplitude of these harmonics. In Knobeler and Wanczek (1993) it was shown, that in the case of *α *=* *π/*3* (the detection electrode of 2*α *=* *120°), the third harmonic is equal to zero. Thus, traps with detection and excitation electrodes of 120° and 60° correspondingly can eliminate the third harmonic in the spectrum.

We should note that those “parasitic” harmonics could be useful as well. The cyclotron radius of an ion cloud can be determined using the ratio between the first and third harmonic terms of the analytical expression and those harmonic terms are functions of the radius (Grosshans & Marshall, 1992). Also, it is useful to use higher harmonics for higher resolving power, as it is described below.

#### 2.3.2 Dipolar detection at harmonics and detection by multiple electrodes

Detection on higher‐order harmonics was used to increase the resolving power by detecting higher frequencies (Pan et al., 1987). Resolving power is proportional to the frequency of the detected signal and the harmonics frequencies are higher than the main frequency by a multiplication factor equal to the harmonic number.

**Figure 2:**
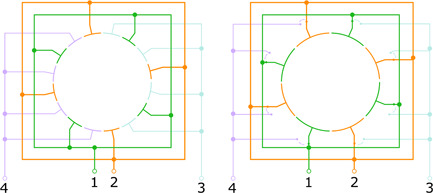
Left: Schematic of an ion cyclotron resonance (ICR) cell with eight excitation and eight detection electrodes, showing how they are connected electrically: 1,2: detection circuit terminals; 3,4: excitation circuit terminals (redrawn from Nikolaev et al. (1990)). Right: Cross‐section of a similar ICR cell where switches are installed so as to quickly reconnect the electrode assemblies from the excitation to detection circuits. This design allows reducing the number of electrodes by a factor of 2 [Color figure can be viewed at wileyonlinelibrary.com]

Another method to increase the detected frequency was proposed by Nikolaev's group in 1985 and demonstrated experimentally in Nikolaev et al. (1985, 1990). In this method, a multiple electrode setup was used, as shown in Figure 2.

In Figure 2, the general schematics of such a cell with four pairs of detection electrodes is presented. Electrodes of the same type are connected together, they are organized into two ensembles of electrodes which are used for the detection of the signal, that is, the difference of potentials between the two ensembles of electrodes. When an ion cloud moves along the cyclotron trajectory of a relatively large radius, it induces a potential difference between the nearest adjacent electrodes, which is equal to that between the two ensembles. Thus, the ion will induce a signal with a period of *n*/2 times smaller (where *n* is the number of electrodes in one circle) than the period of the fundamental cyclotron motion and the frequency of the induced signal will be *n* times larger than the cyclotron frequency.

In such a cell, the signal will be proportional to ∆*Q*

(15) $$\Delta Q=-q\left(\sum_{n=0}^{\infty }{\left(\frac{\rho }{R}\right)}^{4n+2}\frac{4\sin\left(\left(4n+2\right)\alpha \right)}{\left(4n+2\right)\pi }\cos\left(\left(4n+2\right)\varphi \right)\right).$$

By comparing this equation with Equation (13) it can be seen, that in this case only harmonics 2*ω*_+_, 6*ω*_+_, 10*ω*_+_, … remain in the signal (if the center of the cyclotron orbit coincides with the geometrical center of the cell). Note that the calculations above were done for a four detection electrode configuration, which is different from the one shown in Figure 2, where an eight‐electrode configuration is presented, for which the main harmonic would be 4*ω*_+_.

## 3 EVOLUTION OF FT‐ICR TRAPS

To be able to characterize every FT‐ICR trap, we have created a 3D visual image based on the description, given in the articles in which the trap was presented. These images are for illustration purposes only—we intentionally deformed the traps to make their design features more visible. For all cell images, unless otherwise indicated, red color indicates the trapping electrodes, blue the excitation, green the detection, and yellow the compensating electrodes. In all pictures, the *z* axis, which corresponds to the direction of the magnetic field *B*, is oriented from the front‐left to rear‐right (through the centers of the end‐cap electrodes in the closed cells).

All pictures were drawn by the following steps:

• A three‐dimensional model of the trap electrodes was created based on the descriptions (that can be found in the articles) using Python 3.7 programming language. Each voxel has the color corresponding to the specific electrode type (excitation, trapping, etc.), to which this voxel belongs;
• Using the Depth‐First Search algorithm (Tarjan, 1972), the electrodes themselves were formed from the voxels;
• Each electrode was moved a bit away from its original position for better visibility;
• The obtained 3D model was visualized by ipyvolume (Breddels, 2019).
  Second, for every trap the field distribution *ϕ*(*ρ*, *z*) averaged over cyclotron motion is shown. Also in the same figure, the position of the electrodes on a cross‐section (the plane *x *= 0) is shown. The electrodes are colored in the same way as in the 3D model. Note that the position of the electrodes coincides with the equipotentials only in the traps with cylindrical symmetry. For others—like cubic or dynamically harmonized cell—the equipotentials of the averaged *ϕ* should not match the electrode surfaces in the *x* = 0 plane.
• The three‐dimensional model was again created with Python 3.7;
• The electric field distribution inside the trap was calculated using the SIMION 8.1 program (Dahl, 2000);
• The field was averaged over the cyclotron motion trajectory, that is equivalent to averaging over a full *θ* angle range in cylindrical coordinates, so the averaged potential *ϕ*(*ρ*, *z*) was found;
  Third, for each simulated trap, the coefficients from Equation (7) were found assuming *ρ* and *z* are normalized by a characteristic distance *d* (Gabrielse, 1983). By default $d^{2}=\frac{1}{2}{(z}_{0}^{2}+\frac{1}{2}\rho_{0}^{2})$ where *z*_0_ and *ρ*_0_ are characteristic distances in the *z* and *x,y* directions.
  Finally, a rough estimate of comet formation time was done.
  This estimation assumes that ions in the ion cloud lose their coherence when the difference in polar angles between the ions with the maximum and minimum *ω*_+_ from Equation (9) equals 2π. Thus,
(16) $$T_{\mathrm{comet}\;\mathrm{formation}}=\frac{2\pi }{\max_{r,z}\omega_{+}-\min_{r,z}\omega_{+}}.$$
  The geometrical parameters of the ion cloud were taken from (Vladimirov et al., 2012). In this article, the simulation of ion motion inside a cubic trap with full‐length *D = *25.4 mm, trapping voltage *V*_trap_ = 1 V and magnetic field *B *=* *7* *T was done. In FT‐ICR trap simulations, we tuned the free parameters in such a way, so that the *A*_20_ field coefficient at *Y*_20_ would be the same as in the described cubic trap, assuming that in this case, the ion cloud parameters would be the same as well.
  Note that to compare the different types of traps with the cubic one, we kept the diameter of the simulated traps in most cases equal to the length of the cubic cell's edge (*D* = 25.4 mm or 1 in.). In the actual cells used in real life, the dimensions are twice as large. Due to the linearity of electrostatics, the field in larger traps should have the same functional form as in the smaller ones but with proportionately smaller values.
• The averaged electric field distribution was found for each trap as described above;
• The coefficients *A*_20_, *A*_40_, *A*_60_ were calculated from Equation (7) using the least‐squares method in the ion flight region (it was assumed that *R*_max_ = *z*_max_ = 10 mm);
• *ω*_+_ was calculated from Equation (9). The following geometry parameters of the cloud were used: the radius of the cloud *r*_cloud_ = 2 mm, the radius to which the ion cloud was exited *r*_excitation_ = 6 mm, half‐length of the cloud along the *z* axis *z*_cloud_ = 4 × *r*_cloud_ = 8 mm. The following parameters were fixed for all simulations: the magnetic field *B* = 7 T and *m*/*z* ratio = 500, in which *m* is ion mass (Da) and *z* is the number of elementary charges per ion.
  A note about the presented spherical harmonic coefficients and time of comet formation: The coefficients A_20_, A_40_, and A_60_ are presented for all traps under discussion. We kept the same value of the A_20_ coefficient for all traps in the calculation of the time of comet formation. But as one can be seen in Table 2 below these coefficients are slightly different for different traps. This happens because in the table they are dimensionless. These values were obtained by normalization (dividing) of both ρ and z on a characteristic length d. Meanwhile, to estimate the time of comet formation, the same dimensional coefficient A_20_, was used for all traps. By doing so, the electric potential distribution in absolute coordinates was kept the same (in the first order of approximation). This is important because one set of absolute (not normalized) geometrical parameters of the ion cloud was used in all estimations of the time of comet formation. The dimensionless coefficient A_20_ is related to the dimensional coefficient A_20_ “as A_20_ = A_20_” × d^2^.
*The code for generating the geometry of the traps is available at* https://github.com/Lavton/ft_icr_traps_calculation.

**Figure 3:**
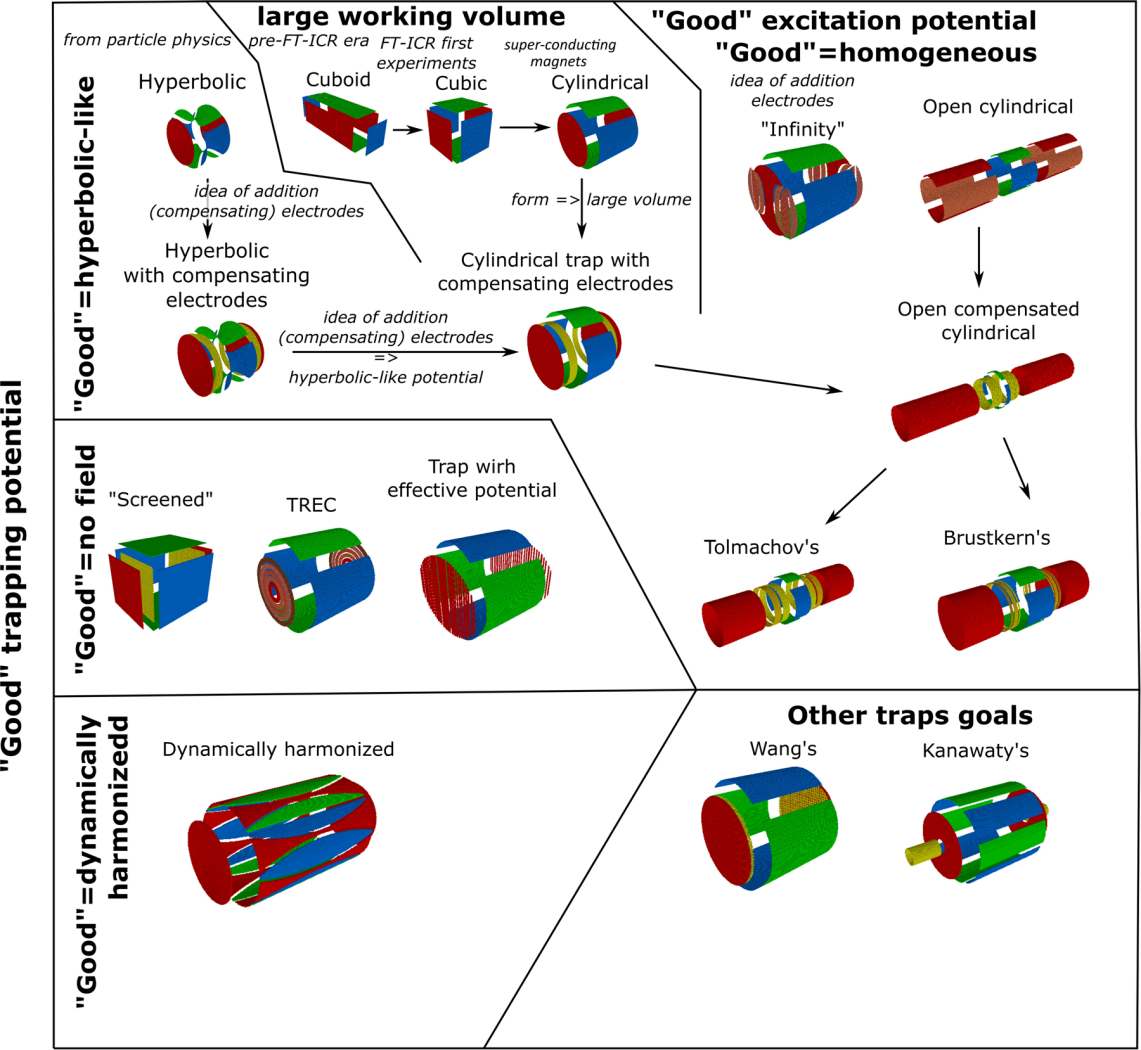
Geometries of different Fourier‐transform ion cyclotron resonance (FT‐ICR) cells and the connection of ideas behind their designs [Color figure can be viewed at wileyonlinelibrary.com]

To describe different traps, we mostly tried to follow their chronological order. But more important is to show the connection of ideas behind these traps. This connection is not always chronological. A map of these connections is presented in Figure 3. The sequence for the description of the different traps is the following: We describe the first group of hyperbolic traps and traps containing compensating electrodes with the trapping field distribution close to hyperbolic; the second group of traps consists of the cuboid, cubic, and cylindrical traps; the third group are the “infinity cell” and open‐cell geometries; and the final group consists of the open cell with compensating electrodes as a synthesis of a closed compensated cell.

Figure 4The design of different Fourier‐transform ion cyclotron resonance cells. Each cell is presented by two figures: a 3D model of the trap (left) and the distribution of the potential averaged over the polar angle (right). Additionally, in the right figures, contours of the electrodes of the trap (in cross‐section x = 0) are superimposed on the figure showing the distribution of the potential. The colors of these contours are the same as the colors of the electrodes on the corresponding 3D image. The abscissa axis in the figure corresponds to the z coordinate of the cell (in mm), the ordinate axis corresponds to the radial coordinate counting from the center of the cell (in mm). The color bar connects the color with the value of the potential. Presented traps: (A) hyperbolic trap, (B) hyperbolic trap with compensating electrodes, (C) cuboid trap, (D) cubic trap, (E) cylindrical trap, (F) cylindrical trap with compensating electrodes, (G) open cylindrical trap with compensating electrodes, (H) trap with compensating electrodes proposed by Tolmachov et al., (I) trap with compensating electrodes proposed by Brustkern et al., (J) the “trapping ring electrode cell,” (K) the dynamically harmonized cell or paracell [Color figure can be viewed at wileyonlinelibrary.com]

**Figure:**
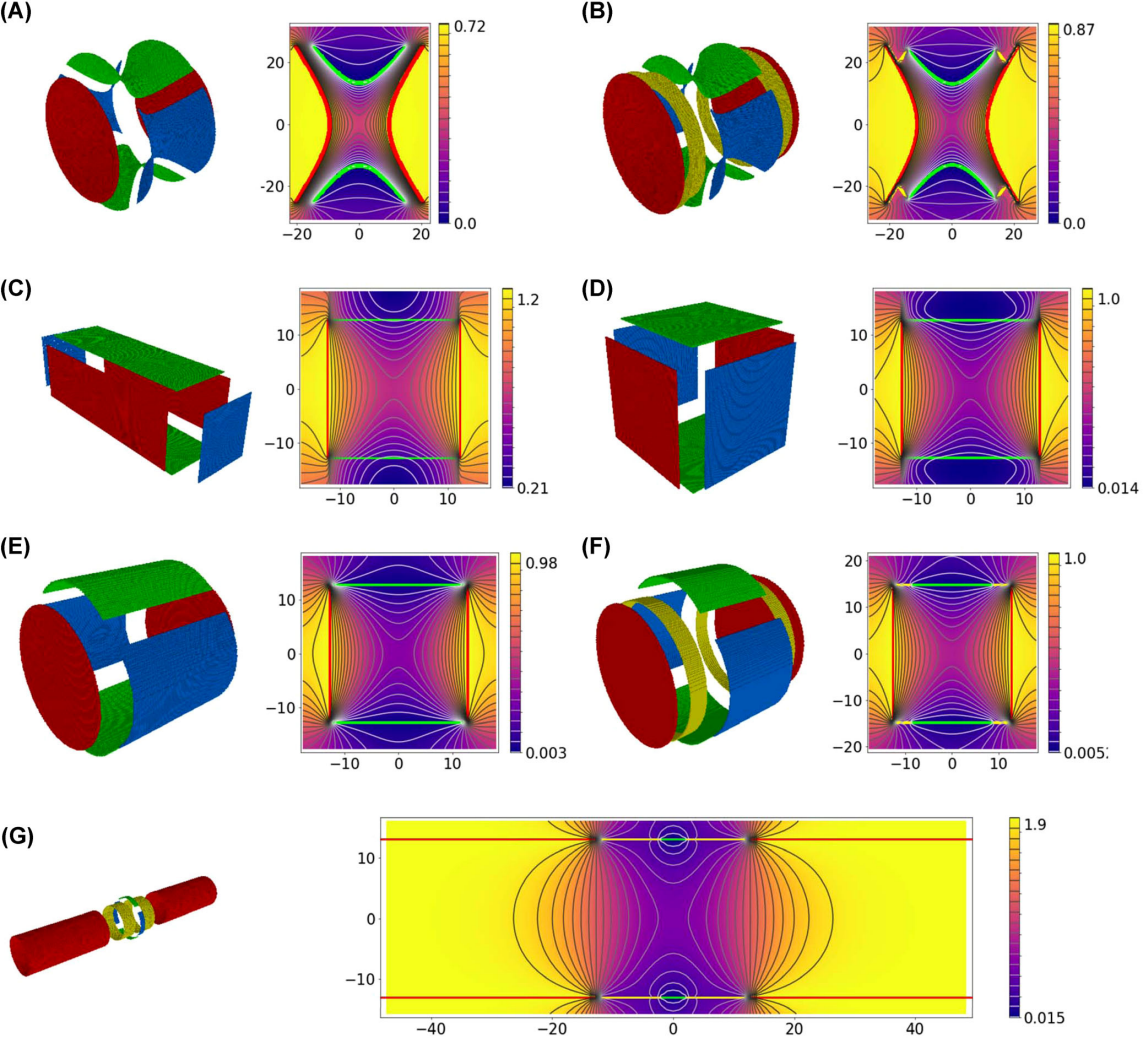

**Figure:**
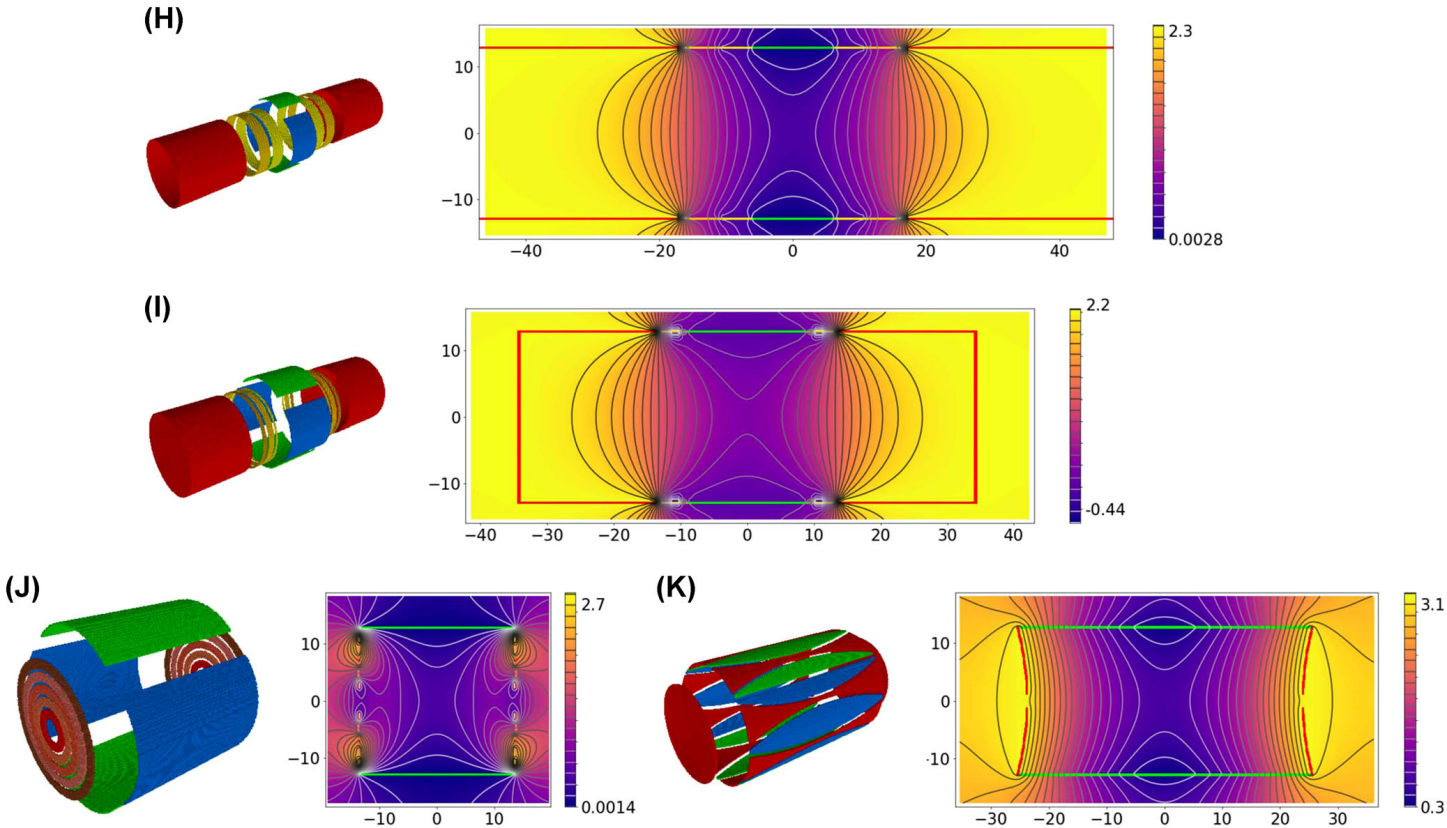

### 3.1 Hyperbolic trap

#### 3.1.1 Described in Van Dyck et al. (1976)

The hyperbolic trap, originally named the Penning trap by H. G. Dehmelt, was introduced in 1959 (Dehmelt, 1989) long before the FT‐ICR method was invented. The surfaces of the trap electrodes follow the equipotentials of a standard hyperbolic potential distribution for such traps (Equation 2; Guan & Marshall, 1995):

(17) $$\begin{array}{c} \phi (x,y,z){|}_{\mathrm{trap}}=\alpha \left(z^{2}-\frac{\rho^{2}}{2}\right)=U_{0},\\ \phi (x,y,z){|}_{\mathrm{ext}\&\det}=\alpha \left(z^{2}-\frac{\rho^{2}}{2}\right)=0. \end{array}$$

So a trap, built in accordance with these equations, will have the following equation for its electrode surfaces (Figure 4A):

(18) $$\begin{array}{llll} 2z^{2}-\rho^{2} & = & 2z_{0}^{2} & (\mathrm{endcap}),\\ \rho^{2}-2z^{2} & = & R^{2} & (\mathrm{ring}). \end{array}$$

The constants *R* and *z*_0_ are the minimum radial and axial distances to the ring and endcap electrodes from the center of the trap, respectively.

The trap is presented schematically in Figure 4A. It was originally used in experimental physics, not mass spectrometry. Theoretically, the trap has an ideal hyperbolic trapping potential distribution. In practice, the main problem is that Equation (18) is valid for infinitely long electrodes, but experimental traps are truncated to a finite size *ρ* ≤ *R*_max_. This leads to a distortion in the potential distribution. The trap can be modified to reduce this distortion: by increasing the distance between the endcaps (Louris et al., 1992) or by modifying the shape of the electrodes (Franzen, 1991). Also, the distortion may be reduced more accurately by introducing correction electrodes, as described below.

### 3.2 Hyperbolic trap with compensating electrodes

#### 3.2.1 Described in Van Dyck et al. (1976) and Gabrielse (1983)

Compensating electrodes were added to the hyperbolic trap to create a field configuration, for which spherical harmonics >*Y*_20_ (especially the largest “parasitic” spherical harmonics—*Y*_40_) are reduced. We will later also discuss the possibility to improve other traps using this approach.

It is quite obvious that for practically all hyperbolic trap configurations (with different ratio *R*/*z*_0_) the trapping and compensation voltages may be selected in such a way, that the coefficient *A*_40_ at *Y*_40_ would be nullified in a certain volume. But a problem arises: Reduction of *A*_40_ by changing the voltage on the compensating electrodes, also requires to change the *A*_20_ coefficient, and, thus, the rotation (reduced cyclotron) frequency. It is easier to use a trap, where the trapping voltage *V*_trap_ tunes *A*_20_, while the compensation voltage *V*_c_ influences only the *A*_40_ coefficient, but not *A*_20_. Such traps are called “orthogonalized.” This is especially important because the holes in the cap electrodes and misalignments in the electrode positions will change the value of *A*_40_ from its theoretical value, thus it will be necessary to be tuned manually during the experiment. Gabrielse has shown (Gabrielse, 1983) that for a specific *R*/*z*_0_ (where *R* is the distance to the ring, *z*_0_ is the distance to the end‐cap from the center of the trap), the trap becomes orthogonalized.

To calculate the geometry of the trap in which *V*_c_ affects only *A*_40_ the potential *ϕ* inside the trap should be presented in the form:

(19) $$\phi =V_{\mathrm{trap}}\phi_{0}+V_{c}\phi_{c}.$$

It can be also presented as a spherical harmonic decomposition as follows:

(20) $$\phi ={\frac{1}{2}V}_{\mathrm{trap}}\sum_{k=0}^{\infty }A_{k}\frac{r^{k}}{d^{k}}P_{k}(\cos\theta ),$$

where *d* ^2^ *= *(1/2) (*z*_0_^2^ + (1/2) *R* ^2^).

Each *A*_k_ may be split into two parts: a part independent of *V*_c_ and one linearly dependent on it (think about it as the Taylor decomposition) as follows:

(21) $$A_{k}=A_{k}^{\left(0\right)}+\left[V_{\mathrm{trap}}\frac{\partial A_{k}}{\partial V_{c}}\right]\frac{V_{c}}{V_{\mathrm{trap}}}.$$

The second term, which depends on *V*_c_, may be used to cancel out the first one and thus to eliminate *A*_k_.

Because the goal is to make ∂*A*_2_/∂*V*_c_ = 0, while ∂*A*_4_/∂*V*_c_ ≠ 0, a quality factor *γ* is introduced as follows:

(22) $$\gamma =\frac{V_{\mathrm{trap}}\partial A_{2}/\partial V_{c}}{V_{\mathrm{trap}}\partial A_{4}/\partial V_{c}}.$$

The goal may now be reformulated as “to find such a configuration for which *γ* will be as close to zero as possible.”

Using the relaxation method for numerical Laplace calculations, Gabrielse has shown that starting from the distance of the compensating electrode from the center of the trap *r*_c_ = 2 *d*, *γ* can be made close to zero independently from a particular *r*_c_ for any electrode shape. This optimal configuration for the hyperbolic trap is reached when *R* = 1.16 *z*_0_. This ratio was used for modeling; the results are shown in Figure 4B.

### 3.3 Cuboid trap

#### 3.3.1 Described in McIver (1970) and Sharp et al. (1972)

The first FT‐ICR experiments were performed with a cuboid cell of the following dimensions 2.54 × 2.54 × 7.62 cm, as described in Comisarow (1981). Such a cell was inherited from the pre‐FT era since 1970 (McIver, 1970). It had long trapping electrodes, as shown in Figure 4C. Dimensions of the trap were fitted to the size of the gap between electromagnet poles.

The analytical solution for the potential inside such a cuboid trap was given in Sharp et al., (1972):

(23) $$\begin{array}{l} V=V_{0}+16\pi^{-2}\left(V_{t}-V_{0}\right)\\ \;\times \sum_{m=0}^{\infty }\sum_{n=0}^{\infty }\left[\frac{\cos h\left(\frac{k_{mn}\pi z}{c}\right){\left(-1\right)}^{m+n}}{\cos h\left(\frac{k_{\mathrm{mn}}\pi }{2}\right)}\right.\\ \;\left.\times \frac{\cos\left[\frac{\left(2m+1\right)\pi x}{a}\right]\cos\left[\frac{\left(2n+1\right)\pi y}{b}\right]}{\left(2m+1)(2n+1\right)}\right], \end{array}$$

where *k*_nm_ = ((2*m* + 1)*c*/*a* ^2^ + (2*n* + 1)*c*/*b* ^2^)^1/2^; *V*_t_ is the trapping voltage; *V*_0_ is the voltage of other electrodes; *a*, *b*, *c* are the dimensions of the trap in the *x*, *y*, and *z* direction, respectively. Ion motion in the cell was analyzed in Sharp et al., (1972).

### 3.4 Cubic trap

#### 3.4.1 Described in Comisarow (1981)

The cubic cell (Figure 4D) replaced the cuboid one soon after the FT‐ICR method was introduced. It was found that the resolution of the instrument with the cubic trap was 2–4 times higher than in the case of the cuboid one (the time of comet formation in our simulations does not show this advantage and it is likely that there was another reason for this like better confinement of ions in the homogeneous region of the magnetic field), also it was more convenient and had greater reliability, as described in Comisarow (1981).

The potential inside the cubic trap can be calculated from Equation (23) by putting *a *=* b *=* c*.

For this cell, it is possible to calculate the field distribution when excitation voltage is applied to the excitation electrodes (Guan & Marshall, 1995; Rempel et al., 1986):

(24) $$\begin{array}{l} \phi_{\mathrm{dipole},\mathrm{ext}}=V_{\mathrm{dipolar}}\times \left(\frac{\beta_{1}x}{a}+\frac{\beta_{2}x}{a^{3}}\left(\frac{2}{3}x^{2}-y^{2}-z^{2}\right)\right)\\ \;+O(x^{5}+y^{5}+z^{5}), \end{array}$$

with the numerical coefficients *β*_1_ = 0.721, *β*_2_ = 2.366, *V*_dipolar_ is the voltage between two electrodes, *O* (here and later) is the “big O notation”—represents that all other terms are equal or higher order in *x*, *y*, and *z*. Here, the *O*(*x* ^5^ + *y* ^5^ + *z* ^5^) represents terms of the fifth order and higher orders in *x*, *y*, and/or *z*.

### 3.5 Cylindrical trap

#### 3.5.1 Described in Comisarow and Marshall (1976), Kofel et al. (1986), and Lee et al. (1980)

As discussed in Section 1, the transition from the cubic to cylindrical cells was mostly due to technical reasons—FT‐ICR mass spectrometers started to use superconducting magnets with a cylindrical geometry, so a cylindrical trap allowed to use the maximum volume with high magnetic homogeneity (Elkind et al., 1988; Figure 4E).

The analytical solutions for trapping and dipolar‐excitation electric field potentials were given in Kofel et al. (1986) as follows:

For the case, when the only trapping potential exists (no excitation voltage is applied), the analytical solution is

(25) $$V=V_{\mathrm{trap}}\left[1-\sum_{k=1}^{\infty }\frac{4\sin\left(\frac{k\pi }{2}\right)}{\pi kI_{0}\left(\gamma_{k}R\right)}I_{0}\left(\gamma_{k}\rho \right)\cos{(\gamma }_{k}z)\right],$$

where *I*_m_ is the modified Bessel function, *γ*_k_ = *k*π/*z*_0_, *V*_trap_ is the trapping voltage, *R* is the radius of the cell, *z*_0_ is the half‐length of the cell;

And in case of dipolar excitation the solution is

(26) $$\begin{array}{l} V=V_{E}\sum_{m=1}^{\infty }\sum_{k=1}^{\infty }\frac{16\sin^{2}\left(\frac{m\pi }{2}\right)\sin\left(\frac{m\pi }{4}\right)\sin\left(\frac{k\pi }{2}\right)}{\pi^{2}\mathrm{mk}I_{m}\left(\gamma_{k}R\right)}\\ \;I_{m}\left(\gamma_{k}\rho \right)\cos\left(m\varphi \right)\cos\left(\gamma_{k}z\right). \end{array}$$

Near the center of the cell, the equation for trapping potential distribution becomes (Guan & Marshall, 1995):

(27) $$V=V_{\mathrm{trap}}\left[\gamma \prime \prime -\frac{\alpha \prime \prime }{2R^{2}}(x^{2}+y^{2}-2z^{2})\right]+O(x^{4}+y^{4}+z^{4}),$$

where for the case *R *= *z*_0_, the numerical coefficients are γ″ = 0.2787, α″ **=* *2.8404. For an arbitrary aspect ratio, the calculations can be found in Kofel et al. (1986). So, the field distribution is approximately hyperbolic at the center of the trap.

And for the dipolar excitation, the potential distribution is given by the following equation:

(28) $$\begin{array}{l} \phi_{\mathrm{dipole},\mathrm{ext}}=V_{\mathrm{dipole}}\left[\frac{\beta_{1}x}{R}+\frac{\beta_{2}x}{2R^{3}}\left(x^{2}+y^{2}-4z^{2}\right)\right]\\ \;+O(x^{5}+x^{5}+z^{5}), \end{array}$$

where *β*_1_, *β*_2_ are some numerical coefficients that can be found in Kofel et al. (1986) for an arbitrary aspect ratio.

### 3.6 Cylindrical trap with compensating electrodes

#### 3.6.1 Described in Gabrielse and MacKintosh (1984)

The main problem with the cylindrical trap is the inharmonicity of the trapping electric field. Three modes of motion—cyclotron, magnetron, and axial—are not separated because the electric potential does not exactly fit the ideal potential (Equation 2). This leads to comet formation and peak broadening.

Returning to the hyperbolic trap, we should say that in comparison with a cylindrical one, it has at least three disadvantages:

- It was difficult at those times to manufacture electrodes with such a complicated shape comparing to a cylindrical one, and even nowadays, production of cylindrical cells is much easier;
- It is not possible to provide a homogeneous excitation field;
- The main disadvantage is that the working volume of a hyperbolic trap is much smaller than that in a cylindrical one. The number of ions that could be trapped without ion–ion interactions is small and the dynamic range of the cell is limited.

There are two general ways to create the needed hyperbolic potential distribution, as noted in Guan & Marshall (1995).

**Figure 5:**
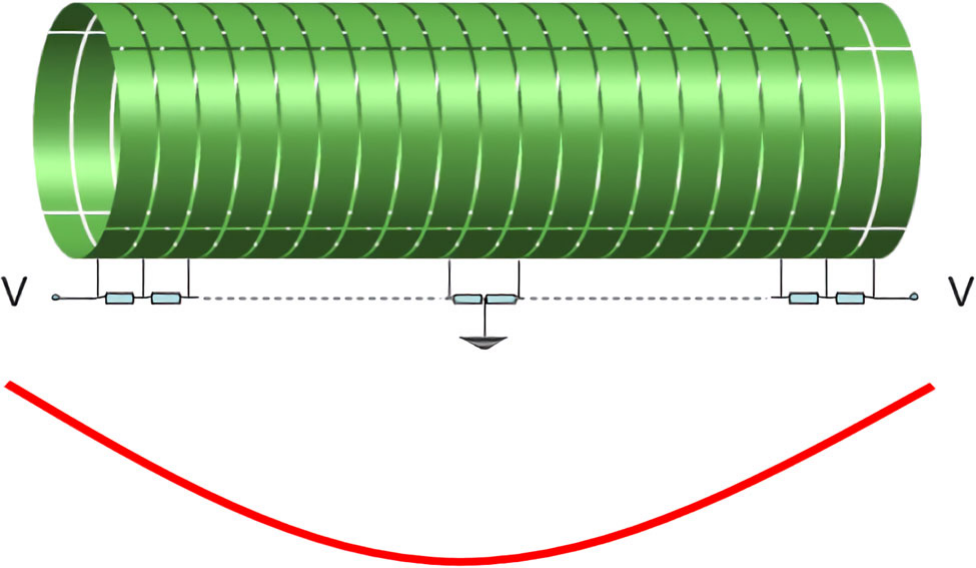
An example of a multielectrode configuration (upper), that provides a ~*z* ^2^ trapping potential (lower) [Color figure can be viewed at wileyonlinelibrary.com]

The first of them, implemented in a hyperbolic trap, consists of using a set of electrodes (three in the case of a hyperbolic trap), the surfaces of which coincide with the hyperbolic equipotentials. The second method of designing cells is used when we have a need for some special geometry of the entire assembly of cells, say, a cylinder (Figure 5) in the case of a dynamically harmonized cell and an open cylinder in the case of compensated cells. In this approach, the surface of the cylinder is segmented into separate electrodes (for excitation, detection, and capture).

The potential on these electrodes should be such that the potential distribution inside the cell becomes as close as possible to the hyperbolic one. So, in the first approach, the potential on the future electrodes is first fixed, and then the geometry of these electrodes is determined; and in the second approach, the position of the electrodes is first fixed, and then their shapes and potentials on them are found. The second approach has been implemented in the cells discussed below.

In Guan & Marshall (1995), the solution for the electric potential for cubic and cylindrical cells in the second approach was given as follows:

(29) $$\phi (x,y,z){|}_{x=\frac{a}{2}}=\alpha \left(z^{2}-\frac{y^{2}}{2}\right)+\frac{\alpha a^{2}}{8}+\chi ,\;\mathrm{cubic}\;\mathrm{trap};$$

(30) $$\phi (x,y,z){|}_{z=\frac{a}{2}}=-\alpha \frac{x^{2}+y^{2}}{2}+\frac{\alpha a^{2}}{4}+\chi ,\;\mathrm{cubic}\;\mathrm{trap}\;;$$

(31) $$\phi (x,y,z){|}_{\rho =\rho_{0}}=\alpha z^{2},\;\mathrm{cylindrical}\;\mathrm{trap};$$

where *α* and *χ* are the same as in Equation (2), *a* is the length of the trap.

**Figure 6:**
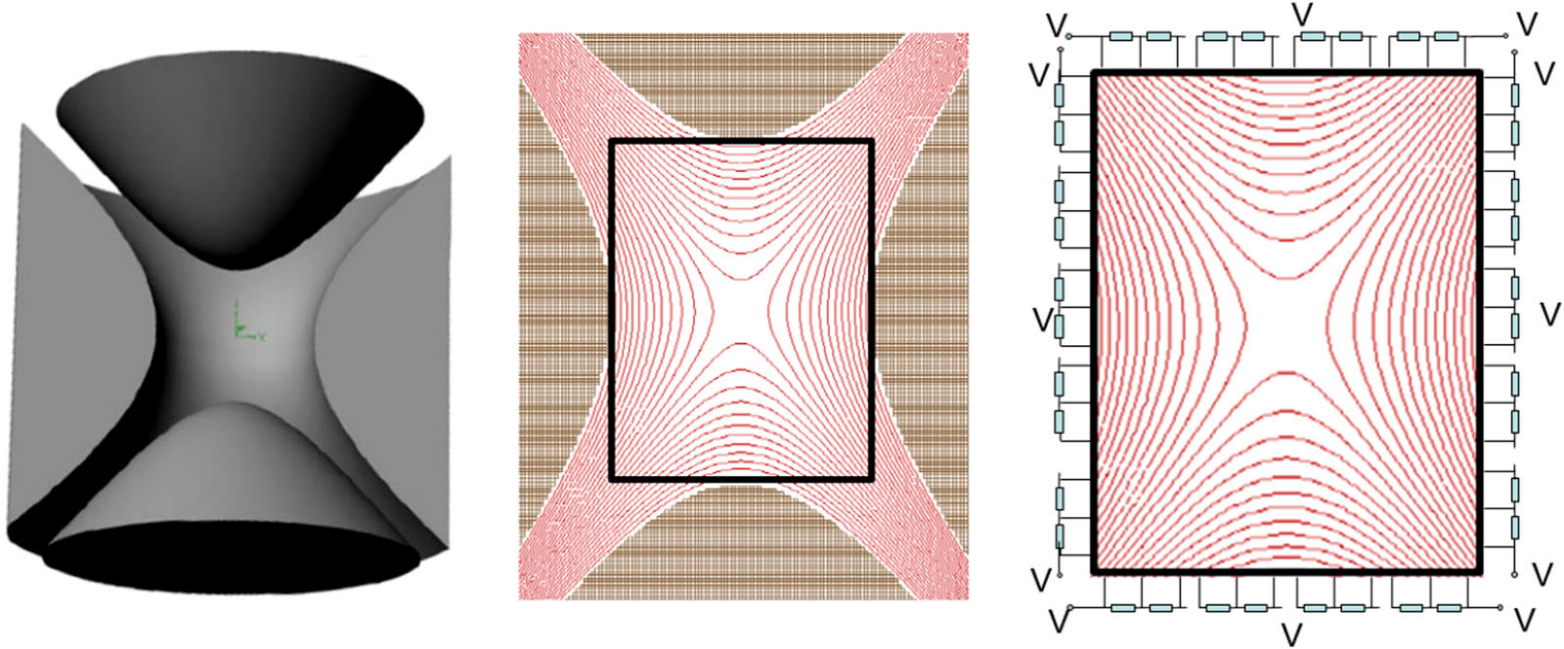
The construction of a trap with a simple geometry and an ideal potential distribution: From the hyperbolic trap (left) we get the potential distribution inside the inscribed cylinder (in the middle) and create a new trap, that emulates this potential distribution using voltage dividers (right) [Color figure can be viewed at wileyonlinelibrary.com]

By splitting the electrodes into segments using these equations, the electric potential may be as close to the ideal one as wanted (see Figures 5 and 6). In practice, the number of electrodes is limited by manufacturing difficulties: A big number of electrodes leads to a very complicated structure with voltage dividers containing a large number of resistors in a vacuum (i.e., not practical). Still, this approach with a limited number of electrodes is implemented in several cylindrical traps, which differ from each other by the electrode configuration. In FT‐ICR, this kind of traps is usually named as “compensated cells.” We will not describe in details every trap, but rather only the “milestone” ones.

One of the simplest modifications of the cylindrical trap is the addition of two ring electrodes (one near each end‐cap), as shown in Figure 4F. By applying proper voltages to these electrodes it is possible to obtain a field distribution in the center of the trap close to hyperbolic. For the same reason as in the case for the hyperbolic trap with compensating electrodes, it is better to use a specific trap geometry, in which tuning of the compensation voltage affects only the *A*_40_, but not the *A*_20_ coefficient. The procedure to obtain such a geometry is practically the same as that for the hyperbolic trap. Now, the electric potential distribution may be found analytically (Gabrielse & MacKintosh, 1984).

The electric potential near the center can be presented in a standard way using modified Legendre polynomials.

(32) $$V=\frac{1}{2}V_{\mathrm{trap}}\sum_{k=0,k\;\mathrm{even}}^{\infty }A_{k}{\left(\frac{r}{d}\right)}^{k}P_{k}(\cos\theta ),$$

where *A*_k_ are coefficients, *d* ^2^ **=* *(1/2)((1/2)*R* ^2^ + *z*_0_^2^), *R* is the radius, and *z*_0_ is the half‐length of the trap.

The analytical solution for *V*_0_ ∂*A*_k_/∂*V*_c_ (for details see calculations done for the hyperbolic trap with compensating electrodes) is

(33) $$\begin{array}{l} V_{0}\frac{\partial A_{k}}{\partial V_{c}}=\frac{{\left(-1\right)}^{\frac{k}{2}}}{k!}\frac{\pi^{k-1}}{2^{k-3}}\frac{d^{k}}{z_{0}^{k}}\\ \;\sum_{n=0}^{\infty }\frac{{\left(-1\right)}^{n}{\left(2n+1\right)}^{k-1}2\sin^{2}\left(\frac{1}{2}k_{n}\Delta z_{c}\right)}{J_{0}(ik_{n}R)}, \end{array}$$

where *i* is sqrt(−1), *k*_n_ = (*n* + 1/2)π/*z*_0_, *J*_0_ is the Bessel function, Δ*z*_c_ is the length of the compensating electrodes.

To make the trap orthogonalized, the *R*/*z*_0_ values can be computed using a function of Δ*z*_c_/*z*_0_ in the article (Gabrielse & MacKintosh, 1984).

Still, the time of comet formation is not very long for this trap because *A*_60_ is rather large.

The same technique was implemented for the cubic cell in Rempel et al. (1990).

### 3.7 “Infinity” and open traps

#### 3.7.1 Described in Beu and Laude (1992a, 1992b), Caravatti and Allemann (1991)

The excitation field distribution in the cells, described above, reveal a problem similar to the nonideality of the electric trapping potential.

**Figure 7:**
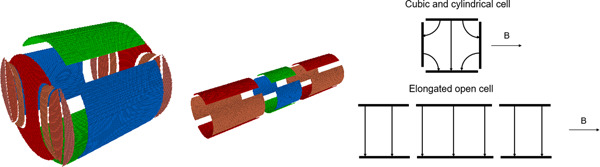
Left—the “infinity cell”; in the middle—the configuration of an open cell with separated trap electrodes; on the right—the distribution pattern of the excitation field in closed and open cells (the square in this scheme represents the cylinder from a side view) [Color figure can be viewed at wileyonlinelibrary.com]

In the closed cylindrical cell, the excitation potential distribution is determined by Equation (28). As mentioned in Section 1, the inhomogeneity of the field along the *z* direction (dependence of the excitation field on the *z* coordinate) leads to the excitation of ions with different axial oscillation amplitudes to different cyclotron radii. In addition, axial oscillations can also be excited during the excitation of cyclotron motion in such a field, leading to the ejection of ions from the cell. In the case of an infinitely long (in the *z* direction) cell, the excitation satisfies Equation (12) with no *z* dependency (Figure 7, right).

Two approaches were applied to emulate an infinitely long trap.

The first one is called the “Infinity Cell” (Caravatti & Allemann, 1991; Figure 7, left). The idea under this trap is very similar to that of the closed compensated cell. Knowing the equation for the field of a true infinitely long trap (Equation 12), flat trapping electrodes are segmented in such a way so that every segment follows the equipotentials of the excitation field for the infinitely long trap. During the excitation process, besides the trapping DC voltage, the excitation RF voltage is applied not only to the main excitation electrodes but also to the segments of the trapping electrodes through a capacitance voltage divider.

The second approach is the so‐called “open” trap configuration (Beu & Laude, 1992b; Figure 7, middle). In this approach, the role of the end‐cap (trapping) electrodes is played by cylindrical trapping electrodes (see figure). These additional electrodes are cut in such a way, that the central excitation electrode has the same polar angle position as the trapping electrode segments. During excitation, these segments of the trapping electrodes are kept at the same RF voltage as the central excitation electrodes. In this way, the excitation field is made independent of *z* in a relatively large area.

(For keeping the visualization simple, we did not split the trapping electrodes in the 3D models in the next subsections. But all open cells usually have these split electrodes).

The other advantage of such traps is open access to the interior of the trap, which makes it easier to introduce microwaves or a laser beam (Gabrielse et al., 1989).

The distribution of the trapping potential field in such kinds of cells is described below.

### 3.8 Open compensated cylindrical trap

#### 3.8.1 Described in Gabrielse et al. (1989)

To combine the advantages of the compensated and open cells, an open cylindrical cell with compensating electrodes was created (Figure 4G).

It follows the same logic as the closed compensated trap. One also can find the trap configuration, in which the compensating voltage *V*_c_ will not affect *A*_40_. But, it was shown that there is a configuration, in which *V*_c_ that nullifies *A*_40_ can nullify *A*_60_ as well. This made the trap much more useful for high‐precision mass measurement experiments (Gabrielse et al., 1989).

The geometrical parameters for this trap are shown in Table 1. Although the simulation does not show that *A*_60_ is equal to zero exactly, it can be attributed to numerical errors. Still, the time of comet formation is an order of magnitude larger in this type of traps than in a standard cylindrical one (Table 2).

**Table 1:** Specific parameters of the simulated geometry of some traps

Trap name	Electrodes color representation and voltage	Geometry parameters
Hyperbolic trap	Red, blue and green are trapping, excitation, and detection electrodes correspondingly	Inner radius of the trap *R* = 1.27 cm, half‐inner length *z*_0_ = *R*/1.16 cm. Both end‐cap and ring electrodes are within *ρ* ≤ *R*_max_ = 2 *R*
	*V*_trap_ = 0.87 V	
Hyperbolic trap with compensating electrodes	Red, blue, green, and yellow are trapping, excitation, detection, and compensating electrodes	Inner radius of the trap *R* = 1.27 cm, half‐inner length *z*_0_=*R*/1.16 cm. The distance from the center to the compensating electrode *r*_c_ = 2 *R*. Both end‐cap and ring electrodes are within *ρ* < = R_max_ =2* *R
	*V*_trap_ = 0.87 V, compensated voltage *V*_c_ = 0.455 V	
Cuboid cell	Red, blue, and green are trapping, excitation, and detection electrodes correspondingly	Lengths 7.62 × 2.54 × 2.54 cm
	*V*_trap_ = 1.2 V	
Cubic cell	Red, blue, and green are trapping, excitation, and detection electrodes correspondingly	The full‐length of the trap *a *= 2.54 cm
	V_trap_ = 1 V	
Cylindrical cell	Red, blue, and green are trapping, excitation, and detection electrodes correspondingly	Radius of the trap *R* = 1.27 cm, half‐length *z*_0_ = 1.27 cm
	*V*_trap_ = 0.988 V	
Cylindrical trap with compensating electrodes	Red, blue, green, and yellow are trapping, excitation, detection, and compensating electrodes correspondingly	Radius of the trap *R *= 1.27 × 1.16 cm, half‐length *z*_0_ = 1.27 cm, the length of compensation a electrode Δ*z* = 0.3 *z*_0_
	*V*_trap_ * * = 1 V, compensation potential *V*_c_ = 0.145 V	
Open cylindrical cell with compensating electrodes	Red, blue, green, and yellow are trapping, excitation, detection, and compensating electrodes correspondingly	Half‐length distance of the area between the trapping electrodes *z*_0_= 1.27 cm, radius of the trap *R* = 1.0239 *z*_0_, length the of compensating electrode Δ*z* = 0.8351 *z*_0_, length of the trapping electrodes *z*_e_ = 4.327 *z*_0_
	*V*_trap_ = 1.949 V, compensation potential *V*_c_ = 0.3235 V	
Trap with compensating electrodes offered by Tolmachev	Red, blue, green, and yellow are trapping, excitation, detection, and compensating electrodes correspondingly	Radius of the trap *R* = 1.27 cm, half‐length of the distance between the trapping electrodes *z*_0_ = 1.3 *R*, length of the compensating electrodes (from the center to border) Δ*z*_1_= 0.4 *R*, Δ*z*_2_ = 0.4 *R*, length of the trapping electrodes *z*_e_ = 2 *R*
	*V*_trap_ = 2.3 V, compensation potentials *V*_c_1_ = 0.1333 *V*_trap_, *V*_c_2_ = 0.3167 *V*_trap_	
Trap with compensating electrodes offered by Brustkern	Red, blue, green, and yellow are trapping, excitation, detection, and compensating electrodes correspondingly	Inner radius of the trap *R* = 1.27 cm, half‐distance of the trapping electrode 21.87 × *a*, length of the compensating electrodes 1.55 × *a*, 5.05 × *a*, 3.05 ×* a*, where *a* = *R*/31.24. Length of the outer electrode *z*_0_ × 4.2/2.8, where *z*_0_ is the length of the trapping and compensating electrodes
	*V*_trap_ = 35 × *k*, *V*_c_0_ = 9.608 × *k*, *V*_c_1_ = *k*, *V*_c_2_= 9.608 × *k*, where *k* = 0.06269 V	
The trapping ring electrode cell	Red, blue, green, and yellow are trapping (different kind), excitation, detection, and compensating electrodes correspondingly	Inner radius of the trap *R* = 1.27 cm, half‐length of the trap *z*_0_ = *R*, number of rings 5, ring width 0.11/*a*, ring gaps 0.039/*a*, where *a* = 1.875
	Ring voltages (from center): 0.2, 1.1, 2.0, 2.4, 2.8 V	
Dynamically harmonized cell or paracell	Red, blue, and green are trapping, excitation, and detection electrodes correspondingly	Radius of the trap *R* = 1.27 cm, half‐length of the trap *z*_0_ = 2 *R*, number of electrodes *N* = 8, fraction of grounded electrodes at *z *= 0, *β*=* *0.9
	*V*_trap_ = 3.06 V	

**Table 2:** The parameters of the simulated electric field distribution in some traps

Trap name	*A*_20_	*A*_40_	*A*_60_	Approximate time of comet formation (s)
Hyperbolic trap	4.3e−01	−2.0e−05	4.5e−06	5.3e+00
Hyperbolic trap with compensating electrodes	4.3e−01	−1.9e−07	2.4e−06	1.8e+01
Cuboid cell	1.9e+00	−2.0e−02	−9.8e−02	3.2e−02
Cubic cell	5.2e−01	4.7e−03	−2.9e−03	2.4e−02
Cylindrical cell	5.3e−01	1.0e−02	−3.6e−03	1.4e−02
Cylindrical trap with compensating electrodes	5.8e−01	2.0e−06	−3.1e−03	3.3e−02
Open cylindrical cell with compensating electrodes	5.3e−01	2.4e−08	−5.9e−04	1.3e−01
Trap with compensating electrodes offered by Tolmachev	7.6e−01	7.2e−05	−1.3e−03	1.8e−01
Trap with compensating electrodes offered by Brustkern	5.8e−01	1.6e−02	−3.5e−03	1.3e−02
Dynamically harmonized cell or paracell	1.6e+00	−4.4e−05	2.6e−04	7.0e+00

### 3.9 Other cylindrical traps with near hyperbolic‐like potential

There are a number of traps that were invented with the same goal: to make the potential inside the trap harmonized. We will not describe all these traps in detail, but rather briefly list some of them.

One of the earliest and famous one, the “Matrix‐Shimmed” ion trap (Jackson et al., 1999), was built as a cubic trap with 5 × 5 electrodes at each side. The voltages on these electrodes (150 electrodes in total) were applied in such a way, that all kinds of electric potentials were close to the ideal one. This cell is based on ideas, described by Equations (29) and (30).

In the year 2000, Bruce et al. (2000) designed a novel cell based on ideas, that are shown in Figure 5.

In Figure 4H, we show the trap offered by Tolmachov et al. (2008) with later improvements made by Kaiser et al. (2011). This example shows that by adding extra electrodes, the potential inside the trap can be made closer to harmonic and, thus, the time of comet formation can be prolonged. In this trap, the goal was to reduce the variation of the *E*_ρ_/*ρ* function. In the improved version (Kaiser et al., 2011), the angle dimensions of the detection electrodes were increased to 120° to reduce the 3*ω*_+_ harmonics during detection.

Brustkern et al. (2008; Figure 4I) offered another type of the compensated trap with a different goal: to reduce the variation of the cyclotron frequency in the cell region near the trap's center.

### 3.10 Traps without an electric field inside

The traps without an electric field inside allow measuring the undisturbed cyclotron frequency (pure, not reduced). It also removes the magnetron motion and the harmonics in the signal caused by it.

The idea is to nullify the trapping electric field inside the area, where the ions are rotating. For this, we need a trap with the field only in the region near the trapping electrodes.

The first attempt to create such a trap has been made by Grosshans et al. (1988). In this study, they just used a very long (6/1) cubic trap with a voltage of 1 V on the trapping electrodes. Thus, they created a very small electric field in the center of the trap.

The next attempt was described in Wang and Marshall (1989) in the so‐called “Screened” trap. It was the cuboid type trap with the addition of a wire grid adjacent to the trapping electrodes inside the trap. The authors have shown that in this trap the detected cyclotron frequency coincides with the cyclotron frequency in an electric field‐free space. The open configuration analog of such a trap was presented in Vartanian et al. (1995).

In 2008, an attempt to flatten the potential distribution inside the cell by use of trapping electrodes segmented into rings with different DC voltages applied to every ring (Weisbrod et al., 2008; Figure 4J) has been made by Bruce's group.

**Figure 8:**
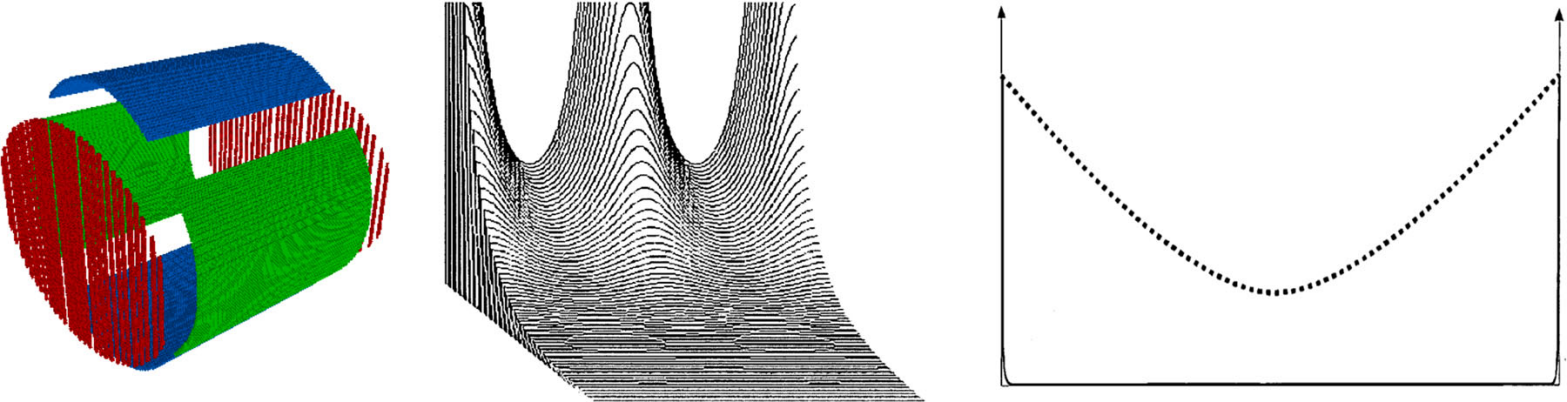
A trap without an electric field in the working area (middle and right pictures are the three‐ and two‐dimensional visualizations of the effective potential from Nikolaev (2005) [Color figure can be viewed at wileyonlinelibrary.com]

What should be mentioned are the attempts to get rid of the electric field inside the trap undertaken by Nikolaev's group (Nikolaev, 2005; Figure 8). A high‐frequency RF voltage applied to an ensemble of wires connected into one used as a trapping electrode was used. An RF voltage with a frequency greater than the cyclotron one creates an effective (or pseudo)‐potential for the ions in the trap (Gaponov & Miller, 1958; Landau & Lifshitz, 1960). The pseudo‐potential penetrates into the cell to a distance that is close (by an order of magnitude) to the distance between adjacent wires.

### 3.11 Dynamically harmonized cell

#### 3.11.1 Described in Boldin and Nikolaev (2011), Kostyukevich et al. (2012), and Lioznov et al. (2019)

The last trap that we will describe in detail, uses a new principle to prevent comet formation. The main idea is to make the potential harmonized not statically (like in compensated traps), but dynamically, by averaging over the cyclotron motion. It is possible because the cyclotron frequency is much bigger than *ω*_z_.

Such a trap prevents comet formation and allows obtaining ultrahigh resolution (Nikolaev et al., 2011). Besides this, the electrodes in this trap are naturally separated into segments, making it easier to use for multielectrode detection.

In this trap, the width of the electrodes depends on *z* as *z* ^2^. Thus, the electrodes should have a leaf‐like shape (Figure 4K). The equation describing the boundary conditions for this trap is the following:

(34) $$\phi {|}_{r=R}=\left\{\begin{array}{c} 0,\;\theta \in \frac{2\pi }{N}n\pm \frac{\beta \pi }{N}\left(1-\frac{z^{2}}{z_{0}^{2}}\right)\\ \phi_{0},\;\theta \in \frac{2\pi }{N}\left(n+\frac{1}{2}\right)\pm \left(\frac{\pi }{N}+\frac{\beta \pi }{N}\left(\frac{z^{2}}{z_{0}^{2}}-1\right)\right) \end{array}\right),$$

where *R* is the radius of the cell, *z*_0_ is its half‐length (*z *= 0 corresponds to the center of the cell), *β* is a coefficient responsible for the width of the grounded (“leaf”) electrodes (more specifically, the ratio between the width of the grounded and nongrounded electrodes in the middle [*z *= 0] of the cell), *N* is the total number of leaf electrodes, *n* = 0, 1, … *N* − 1 is the serial number of the leaf electrode.

And the end‐cap trapping electrode is built based on the following equation:

(35) $$2z^{2}-\rho^{2}=2z_{0}^{2}-R^{2}.$$

In Boldin and Nikolaev (2011), the solution for the averaged potential was found as follows:

(36) $$\hat{\phi }\left(r,z\right)=\phi_{0}-\frac{2\beta \left(1-\frac{z^{2}}{z_{0}^{2}}\right)}{2}\phi_{0}+\frac{\beta \left(1-\frac{r^{2}}{R^{2}}\right)}{2}\frac{R^{2}}{z_{0}^{2}}\phi_{0}.$$

And in Lioznov et al. (2019), the solution for nonaveraged potential was found as well. It is not absolutely strict, but approximate the potential distribution with high accuracy in the whole working volume

(37) $$\begin{array}{l} \phi \left(r,\theta ,z\right)=\frac{\beta \phi_{0}}{2z_{0}^{2}}\left(R^{2}-r^{2}\right)\\ \;+\frac{\phi_{0}}{\pi }\sum_{n=0}^{N-1}{\left(\left(\text{arc}\tan\frac{y-R\sin\theta_{n}}{x-R\cos\theta_{n}}\right)\right|}_{\theta_{n}=\frac{\pi }{N}\left(2n-\frac{\beta z^{2}}{z_{0}^{2}}+\beta \right)}^{\theta_{n}=\frac{\pi }{N}\left(2n+2+\frac{\beta z^{2}}{z_{0}^{2}}-\beta \right)}. \end{array}$$

This trap was modified to have an open geometry in Nikolaev et al. (2020).

The dynamic harmonization method was also implemented in the so‐called “window” cell (Tolmachev et al., 2012). In this cell, dynamic harmonization was achieved by two—inner and outer—ensembles of electrodes. The inner electrodes use a voltage divider, similar to what is shown schematically in Figure 5.

### 3.12 Other trap configurations

**Figure 9:**
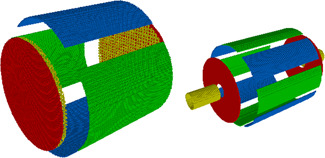
Fourier‐transform ion cyclotron resonance traps for simultaneous detection of positive and negative ions. On the left is a grid electrode system (Wang & Wanczek, 1993); in the middle—a trap with additional cylindrical electrodes (Kanawati & Wanczek, 2007) [Color figure can be viewed at wileyonlinelibrary.com]

For completeness, we should mention, that there were trap designs, that pursuit goals different from trapping field harmonization and excitation field homogenization. For example, there were attempts to create FT‐ICR traps, which would be able to simultaneously trap and detect positive and negative ions. The most successful attempts to create such traps were done by the Wanczek's group (Kanawati & Wanczek, 2007, 2008; Malek & Wanczek, 1996; Y. Wang & Wanczek, 1993). Such traps are shown in Figure 9.

We did not see any new significant ideas in cell design that would give higher resolution and make interpretation of mass spectra easier (reducing harmonics) since the introduction of the dynamically harmonized cell in 2011. There is still some potential for improvement of the dynamically harmonized cell, for example, by making it open to obtain better vacuum inside the cell, or implementing the multielectrode detection method and further decreasing the even harmonics caused by magnetron motion by increasing the precision of electrode production and assembling procedures.

## 4 CONCLUSIONS

In this review, ideas that had the biggest influence on FT‐ICR cell design were described. We tried to follow the evolution from a rather simple cuboid cell through compensated cells to the cell with dynamical harmonization. A further increase in the resolution of FT‐ICR mass‐analyzers can be achieved by increasing the magnetic field and by detecting the signal induced by ions with several pairs of electrodes connected into one (multielectrode detection). This is an old idea offered in 1985 (Nikolaev et al., 1985), implemented for the first time in 1990 (Nikolaev et al., 1990), and only recently came to be used in the FT‐ICR practice. The Bruker scimaX magnetic resonance mass spectrometer uses quadrupole detection to double the frequency and resolution. Several groups have experimentally confirmed this possibility as well (Park et al., 2020; Shaw et al., 2018). This approach is not equivalent to the use of a higher magnetic field because it increases the resolving power only when FT is used for spectra processing. The real resolving power measured as the smallest distance between resolved adjacent peaks in the mass spectrum is limited by the peak coalescence phenomenon and grows with the magnetic field, but stays constant in the case of the increased number of electrodes for multielectrode detection (Nikolaev et al., 2016, 2019). (In case of coalescence, when two ion clouds with different ion masses are merged into one, signal processing cannot separate their frequencies, which are the same physically).

Meanwhile, some of the unresolved questions remain.

First, new FT‐ICR instruments with 15‐ and 21‐T magnets do not show an increase in the resolving power proportional to the magnetic field. This might be due to insufficient vacuum in the system. Vacuum must be improved at least proportionately with the magnetic field because the length of the trajectory that the ions cover during the fixed time of their cyclotron motion is proportional to the magnetic field strength as well. Improved pumping may be achieved either by further improving the pumping system or by changing the cell configuration to make it more open with a smaller surface.

Second, in the case of the multiple electrode detection method, the signal is very sensitive to the quality of the alignment of the cell axis with the direction of the magnetic field. Bad alignment can cause the appearance of spurious harmonics in the spectrum, which complicates the interpretation of mass spectra. To what extent electrode misalignments and accuracy of their manufacturing influence the mass spectra is not yet known. Should they be in the submillimeter or even the micrometer range? The effects of these factors must be carefully investigated both theoretically and experimentally, before spurious harmonics can be eliminated.

Third, it is known that magnetron motion can be excited during the ion capturing event. The dynamics of ion trapping has also not been investigated deeply enough to give a recipe on how to avoid magnetron motion excitation. Another problem is avoiding magnetron motion excitation during excitation of the cyclotron motion. Further research must be done to create such excitation procedures that would not excite the magnetron motion.

Despite these problems, it can be safely assumed that in the next decade the resolution of the top FT‐ICR spectrometers can reach up to 100 million for *m*/*q* 1000 due to further increase in the magnetic field and continuing improvements of the FT‐ICR cell.
